# Task Constraints Affect Mapping From Approximate Number System Estimates to Symbolic Numbers

**DOI:** 10.3389/fpsyg.2018.01801

**Published:** 2018-10-16

**Authors:** Dana L. Chesney, Percival G. Matthews

**Affiliations:** ^1^Department of Psychology, St. John’s University, Jamaica, NY, United States; ^2^Department of Educational Psychology, University of Wisconsin-Madison, Madison, WI, United States

**Keywords:** approximate number system, symbolic number mapping, number-lines, ratios, estimation

## Abstract

The Approximate Number System (ANS) allows individuals to assess nonsymbolic numerical magnitudes (e.g., the number of apples on a tree) without counting. Several prominent theories posit that human understanding of symbolic numbers is based – at least in part – on mapping number symbols (e.g., 14) to their ANS-processed nonsymbolic analogs. Number-line estimation – where participants place numerical values on a bounded number-line – has become a key task used in research on this mapping. However, some research suggests that such number-line estimation tasks are actually proportion judgment tasks, as number-line estimation requires people to estimate the magnitude of the to-be-placed value, relative to set upper and lower endpoints, and thus do not so directly reflect magnitude representations. Here, we extend this work, assessing performance on nonsymbolic tasks that should more directly interface with the ANS. We compared adults’ (*n* = 31) performance when placing nonsymbolic numerosities (dot arrays) on number-lines to their performance with the same stimuli on two other tasks: Free estimation tasks where participants simply estimate the cardinality of dot arrays, and ratio estimation tasks where participants estimate the ratio instantiated by a pair of arrays. We found that performance on these tasks was quite different, with number-line and ratio estimation tasks failing to the show classic psychophysical error patterns of scalar variability seen in the free estimation task. We conclude the constraints of tasks using stimuli that access the ANS lead to considerably different mapping performance and that these differences must be accounted for when evaluating theories of numerical cognition. Additionally, participants showed typical underestimation patterns in the free estimation task, but were quite accurate on the ratio task. We discuss potential implications of these findings for theories regarding the mapping between ANS magnitudes and symbolic numbers.

## Introduction

Humans and many nonhuman animals are equipped with a phylogenetically ancient approximate number system (ANS) that allows them to rapidly enumerate the items in a set without counting (Kaufman et al., 1949; Mechner, 1958; Meck and Church, 1983; Feigenson et al., 2004; Izard and Dehaene, 2008). These findings have led many to conclude that the meanings of symbolic numbers are grounded in part by mapping number symbols (e.g., 5) to their nonsymbolic analogs (e.g., an array of 5 dots) (Nieder and Dehaene, 2009). This obvious symbol-to-referent match is a large part of the appeal of the analog portion of Dehaene (1992) triple code model and of Piazza (2010) hypothesis about the ANS’ role as a neurocognitive start-up tool for number concepts. Although there is substantial disagreement surrounding ANS-as-foundation arguments (e.g., Lyons et al., 2012; De Smedt et al., 2013; Reynvoet and Sasanguie, 2016; Leibovich et al., 2017; Núñez, 2017), this point of view remains widespread.

Number-line estimation – in which participants place numerical values on a bounded number-line – has become a key task used in research on the link between symbolic numbers and numerical magnitudes (Siegler and Opfer, 2003; Whyte and Bull, 2008; Schley and Peters, 2014). Some consider the spacing and precision of number-line placements to directly reflect the spacing and precision of the magnitudes mapped to symbolic numbers (Siegler and Opfer, 2003; Whyte and Bull, 2008). However, this interpretation of number-line performance remains contested. Some researchers (e.g., Barth and Paladino, 2011) argue that number-line tasks are *proportion judgment* tasks as they require people to estimate the magnitudes of the stimuli relative to the endpoints. Prior research indicates such anchored tasks are fundamentally different from tasks for which participants are free to give any response (Banks and Coleman, 1981; Hollands and Dyre, 2000). As such, task demands may influence participants’ mapping responses.

Moreover, there is reason to question the underlying assumption that people can exploit a 1-to-1 map from symbols to their analog numerosities. More than 75 years of research suggest that the vast majority of educated humans cannot accurately make such mappings (Taves, 1941; Kaufman et al., 1949; Indow and Ida, 1977; Krueger, 1984; Izard and Dehaene, 2008; Crollen et al., 2011). In study after study, ANS-based estimations yield under-estimations, and performance varies considerably between participants (Indow and Ida, 1977; Krueger, 1984; Izard and Dehaene, 2008). Given that ANS-based estimation is both inaccurate generally and inconsistent among individuals, it is difficult to see how such a system can be used for grounding symbolic numbers.

Here we seek to clarify principles governing the potential links between ANS-perceived magnitudes and symbolic numbers and how responses based on those links are affected by different task constraints. We investigated how three separate tasks that employ the same sorts of ANS stimuli lead to differences in mapping performance: free estimation, number-line estimation, and ratio estimation.

### Predictions

#### Free Estimation

In free estimation tasks, participants are instructed to give numerical estimates for a range of stimuli whose magnitudes vary on a given dimension, with no given upper bound. This sort of estimation with numerosities has often been described as representing subjective numerical magnitudes in a logarithmic fashion, such that the perceived distance between stimuli is proportional to the logarithm of the ratio between them (e.g., Moyer and Landauer, 1967; Dehaene, 1992). Hence, the perceived difference between 10 and 20 dots is the same as that between 22 and 44, or that between 32 and 64 dots. Izard and Dehaene (2008) offered a model whereby idiosyncrasies in mapping between logarithmically encoded perceived magnitude and actual symbolic numerical responses results in performance that is typically fit by power functions (e.g., Stevens, 1957; Crollen et al., 2011; but see Cordes et al., 2001, for a linear interpretation). Indeed, performance patterns on such unbounded estimations in general – whether involving numerosities or other magnitudes like auditory volume or light intensities – are typically fit by accelerating or decelerating power functions [perceived stimulus intensity = C ^∗^ (Actual stimulus intensity)^B^, where B is the Stevens’ exponent e.g., Stevens, 1957; Indow and Ida, 1977; Krueger, 1984; Crollen et al., 2011].

In the ANS-based free estimation task we use here, participants were asked to provide estimates of the numerosity of nonsymbolic numerical stimuli (dot arrays). We expected unbounded estimation with dot arrays to be characterized by compressive power functions (i.e., Stevens’ exponent < 1), as is consistent with established theory and prior empirical findings (e.g., Stevens, 1957; Crollen et al., 2011). We also expected estimates to exhibit scalar variability (Cordes et al., 2001; Izard and Dehaene, 2008; Crollen et al., 2011). That is, we expected the variability of estimates to increase in proportion to the size of the stimulus, resulting in a constant coefficient of variation (Whalen et al., 1999; Gallistel and Gelman, 2000; Izard and Dehaene, 2008).

#### Number-Line Estimation

**FIGURE 1:**
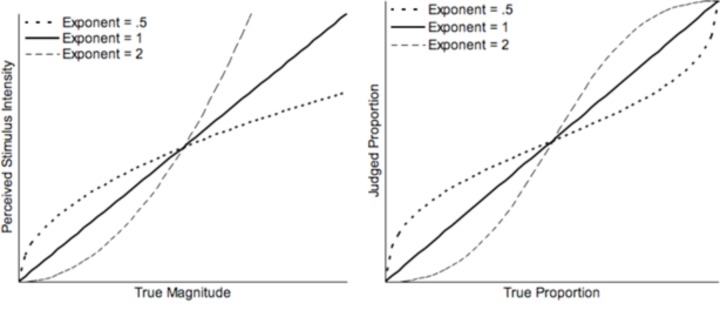
Left: Perceived stimulus intensity as a function of true magnitude as predicted by a power model with exponents of 0.5, 1, or 2. Values are scaled such that the perceived intensity of central magnitudes are equal. Right: Judged proportion as a function of true proportion as predicted by a cyclical power model with exponents of 0.5, 1, or 2. The functions illustrated in these graphs are adapted from Hollands and Dyre (2000).

Our predictions for number line estimation are based on Barth and Paladino (2011) argument that these tasks cannot properly be categorized as free numerical estimation tasks and that they are actually a form of a proportion judgment task. Number line estimation requires that people estimate the magnitude of one stimulus, the to-be-placed value, relative to two other stimuli, the upper and lower endpoints (Spence, 1990; Hollands and Dyre, 2000; Hollands et al., 2002; but see Opfer et al., 2011). For example, when placing 25 on a 0–100 line (whether symbolic or nonsymbolic), it should be 25 units away from 0, and 75 units away from 100. It should therefore be placed at a point corresponding to the proportion between the stimulus and the sum of the stimulus and its complement (25/(25 + 75)), or one fourth of the total length of the line away from 0. No matter what number is estimated, the line must, similarly, be broken into two sections with a constant sum, resulting in a proportion. Spence (1990) offered a cyclical correction to the power model used to describe free estimation that could account for the proportional nature of tasks like number line estimation. This cyclical power model predicts nearly linear performance on number line estimation tasks even given compressive underlying subjective representations of numerical magnitudes (see also Hollands and Dyre, 2000; Hollands et al., 2002; Barth and Paladino, 2011). However, in approaching linearity, cyclical power models show specific patterns of over- and under-estimation for estimates in different segments of the range defined by specific cut points (see **Figure 1**).

Here, we used an ANS-based number-line estimation task. Participants were instructed to estimate the appropriate placement of a nonsymbolic numerical stimulus (a dot array) on a line segment bounded by nonsymbolic numerical anchors at each end. To date, relatively few studies have attempted to use number-line style tasks using nonsymbolic numerosity (dot arrays) in place of symbolic numbers (Anobile et al., 2012; Sasanguie and Reynvoet, 2013; Kim and Opfer, 2015). None of these investigated whether line estimation with dot array stimuli bears signatures of the cyclical power model as might be predicted following Spence (1990) or Hollands and Dyre (2000). We predicted that these tasks would be fit by a cyclical power model and its characteristics: (a) median estimates should be close to the correct value of the stimulus, (b) the standard deviations of the estimates would *not* show scalar variability patterns, but rather would decrease at both end-point anchors and at the midpoint of the line, and (c) participant responses should exhibit a cyclical pattern of over and then under estimation.

#### Ratio Estimation

Here, we used an ANS-based ratio estimation task, asking participants to estimate the ratios instantiated by a pair of nonsymbolic numerical stimuli. Recent research suggests that humans and other animals possess a nonsymbolic ratio processing system (RPS) that is tuned to the magnitudes of nonsymbolically instantiated ratios (Jacob et al., 2012; Matthews and Chesney, 2015; Matthews and Lewis, 2016; Matthews et al., 2016; Bonn and Cantlon, 2017).

Unlike proportion judgment tasks, which are typically conceived of as involving judgment of one portion of the whole relative to the judgment of that portion and its complement (Spence, 1990; Hollands and Dyre, 2000; Hollands et al., 2002; Barth and Paladino, 2011), the part:part ratios used in ratio estimation don’t have the same constraints. Because the physical magnitudes instantiating the high and low anchors vary considerably from trial to trial, the figure-plus-complement logic of the cyclical power model no longer applies. Accordingly, ratio estimation is posited to proceed from a more direct perceptual mechanism (Jacob and Nieder, 2009; Matthews and Chesney, 2015; Lewis et al., 2016) as opposed to the strategy-bound method that results in cyclical performance on line-based proportion judgment tasks (Spence, 1990; Barth and Paladino, 2011; Cohen and Blanc-Goldhammer, 2011). Indeed, single-cell recordings from primates suggest that there are neurons that respond specifically to visuospatially constructed ratios as opposed to the magnitude of either component of a given ratio (Vallentin and Nieder, 2008).

RPS theories posit that humans can extract the magnitudes of ratios made from a variety of different stimuli, and several studies have directly investigated the human ability to process ratios composed of dot arrays (McCrink and Wynn, 2007; Fabbri et al., 2012; Matthews and Chesney, 2015). Past research on direct estimation of nonsymbolic ratios made from dot arrays guide our predictions. For instance, Varey et al. (1990), found approximately linear responses in a task similar to our ratio estimation task. Moreover, when Matthews and Chesney (2015) had participants compare symbolic ratios to nonsymbolic ratios, results indicated that participants mapped nonsymbolic dot ratios to numerical ratios in a linear fashion, albeit with a bias that somewhat inflated the size of the nonsymbolic ratios by a constant factor. Finally, in an unpublished pilot study we conducted, we also found that participants’ average estimates were largely accurate. These behavioral findings have been complemented by single-cell recordings from primates suggesting that there are neurons that respond specifically to visuospatially constructed ratios as opposed to the magnitude of either component of a given ratio (Vallentin and Nieder, 2008).

Thus, we expected a linear relation between participant estimates and actual stimulus values for ratio estimation tasks (as opposed to the curvilinear relations predicted for free estimation and line estimation tasks). Although we also expected the number-line estimation task to yield roughly linear estimates, we expected those results to diverge from ratio estimates. This is because we expected ratio estimation to proceed from a more direct perceptual mechanism (Jacob and Nieder, 2009; Matthews and Chesney, 2015; Lewis et al., 2016) as opposed to the strategy-bound method that results in cyclical performance on line-based proportion judgment tasks (Spence, 1990; Barth and Paladino, 2011; Cohen and Blanc-Goldhammer, 2011). As result, we did not expect to see such strategy-based cyclical bias patterns with the ratio estimation task.

## Materials and Methods

### Participants

Participants were 31 undergraduates (16 female, 26 white, mean age 19.3 years (*SD* = 1.1 years) at a highly selective, private university in the Midwestern United States who participated for course credit in the Psychology Department.

#### Materials and Design

All training and testing stimuli were presented using Superlab 4 software (Cedrus Corporation, 2007) on Apple^®^ iMac 5.1 computers running OS10.6. Each computer had a 17” LCD display with a resolution of 1,440 × 900 pixels and a refresh rate of 60 Hz. These screen dimensions subtended approximately 34° × 22° of visual angle with participants seated ∼60 cm from the screen. Degrees of visual angle are only approximate as no restraints were used to restrict head motion.

#### Dot Array Stimuli

Arrays were composed of black dots on a white background. For each array, dot sizes ranged from 1.3 mm to 9.9 mm in diameter (0.1–0.9°), and the minimum distance between dots was 1 mm (0.1°). Dots were arranged randomly in a 76 × 76 mm (7° × 7°) area, such that all arrays had the same convex hull. It was essential to our design that participants used the ANS to estimate the cardinality of the dot arrays, rather than relying upon counting. Accordingly, the smallest numerosity displayed in a given array was 20 to ensure that other fast enumeration techniques, such as subitizing, could not be employed (see Kaufman et al., 1949; Revkin et al., 2008). The dot arrays in each task ranged in numerosity from 20 to 300 dots. The 17 magnitudes represented were: 20, 40, 60, 80, 100, 120, 140, 150, 160, 180, 200, 220, 240, 260, 280, 290, and 300. Stimuli were presented only briefly (1,500 ms). Brief presentation times have been used successfully to suppress counting in previous work (e.g., Revkin et al., 2008).

**FIGURE 2:**
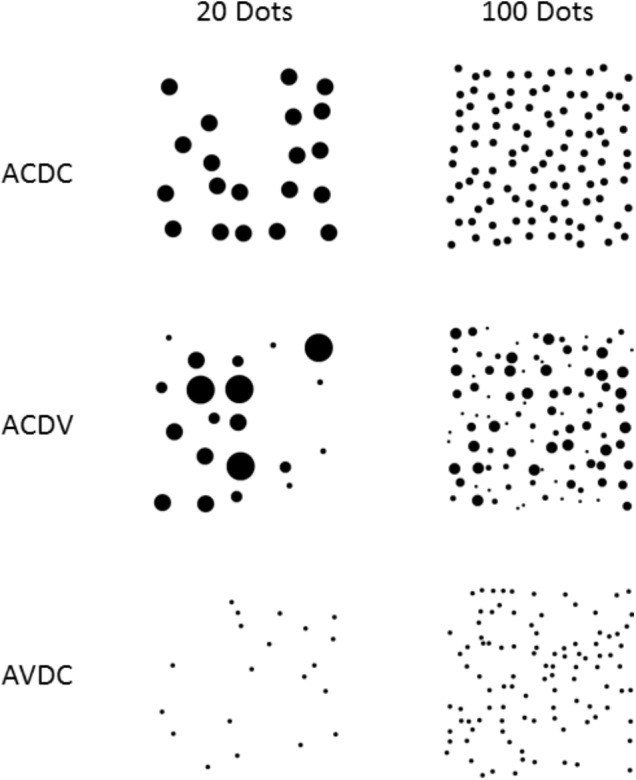
Arrays of 20 and 100 dots in the three continuous extent control conditions: area controlled, dot size controlled (ACDC), area controlled, dot size varied (ACDV), and area varied, dot size controlled (AVDC).

To ensure that nonnumeric features of the arrays would not be consistently related to numerosity, we created three different stimuli for each numerosity, with different controls for individual dot size and summed area (see **Figure 2**). In the *area controlled, dot sizes controlled (ACDC)* arrays, the total surface area was controlled such that all arrays had the same total surface area regardless of dot numerosity, and all dots within any given array were of the same size. As a result, the sizes of individual dots in an array varied inversely and density varied directly with the numerosity of the array. In the *area controlled, dot size varied* arrays (ACDV), total surface area was controlled so that all arrays had the same total surface area regardless of dot numerosity. However, individual dot size varied both within and between arrays, such that the size of a given dot did not precisely correlate with array numerosity. As a result, for these arrays, neither total area nor individual dot size was correlated with numerosity (though the mean dot size of an array was inversely correlated with numerosity). In the *area varied, dot size controlled* (AVDC) arrays, all dots were the same size, regardless of the numerosity. As a result, surface area and density increased linearly with the total numerosity of dots presented. These controls mirror those that have been used in previous studies of numerosity perception (Xu et al., 2005; Hurewitz et al., 2006).

### Procedure

**FIGURE 3:**
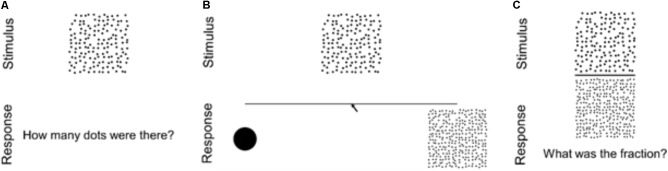
Diagrams of trials in the three estimation conditions: **(A)** free estimation, **(B)** number-line estimation, **(C)** ratio estimation.

Participants first completed the ratio estimation block, followed by the number-line estimation block, and finally the free estimation block (see **Figure 3**). We placed blocks in this order to minimize the likelihood that any block would affect estimation on the subsequent block. Each block began with a set of instructions, using example stimuli that were different from the experimental stimuli. Participants were told that the dot arrays would be presented too quickly for them to count, and that they should “just try to feel out how many dots there are instead of applying a formula.” In all trials, participants pressed a space bar to initiate the trial, then stimuli were briefly presented (1,500 ms), and finally participants were asked to make their responses. If participants did not answer within 15,000 ms, the trial ended automatically. Trial order was randomized within each block. Participants also completed similar tasks involving circle areas, a symbolic number line task, and several mathematics assessments not discussed in this manuscript. We note that, due to experimenter error, one participant completed nearly double the number of trials for each task.

#### Free Estimation

For each trial, a stimulus array was presented for 1,500 ms immediately after the participants initiated the trial. Once the stimulus disappeared, a textbox appeared asking, “How many dots were there?” Participants entered their answers into a text box via keyboard. After responding, they were prompted to hit return to move on to the next trial. Participants completed 51 trials, one for each of the 17 dot numerosities presented in each of the 3 dot array types.

#### Number-Line Estimation

For each trial, participants were shown a “number-line” anchored by one dot on the left and 300 dots on the right. Participants were never told the number of dots on the high anchor. When participants hit the space bar to initiate each trial, the line and anchors appeared. After 1,000 ms elapsed, the stimulus array was presented 25 mm above the center of the line for 1,500 ms. Once the stimulus disappeared, participants used a mouse to indicate the position on the line corresponding to the stimulus numerosity. The line and anchors remained on the screen throughout the duration of each trial. After responding, they were prompted to hit return to move on to the next trial. Participants completed 51 trials, one for each of the 17 dot numerosities presented in each of the 3 dot array types.

#### Ratio Estimation

In ratio estimation trials, participants were instructed to estimate the ratio between the numbers of dots in the two arrays composing each stimulus. Each stimulus was presented for 1,500 ms immediately after the participants initiated a trial. Once the stimulus disappeared, a textbox appeared asking, “What was the fraction?” Participants then typed their answers into a text box via the keyboard. After responding, they were prompted to hit return to move on to the next trial. Participants completed 51 trials, one for each of the 17 dot numerosities in each of the three formats used in the free estimation and number-line estimation blocks, with the 300 dot stimulus of the matching ACDC, ACDV, or AVDC type in the denominator position (e.g., 20 dots/300 dots, 150 dots/300 dots). Additional trials using denominators of other numerosities were also included, however, only the 300 denominator trials are presented in the results here, so as to increase comparability between blocks.

## Results

### Coding

On the free estimation trials, analyses used participants’ raw responses. One outlier (“9101”) was dropped from consideration. Participants’ spatial position responses on the number-line estimation trials were converted to numerical form corresponding to each response’s relative location on a 1–300 linear number-line. For example, a click on the midpoint of the line was coded as a response of 150. Responses on the ratio estimation trials were first converted to decimal format (e.g., ½, 50/100, and 150/300 were all coded as 0.5). Decimal answers (e.g., 0.8) were also accepted. Trials where participants failed to provide a complete ratio (19 trials) or provided values greater than 5/2 (5 trials) were dropped from consideration. Coded values were then multiple by 300 to place them on the same scale as the Free estimation and number-line estimation tasks for the purposes of analysis.

### Analysis

**FIGURE 4:**
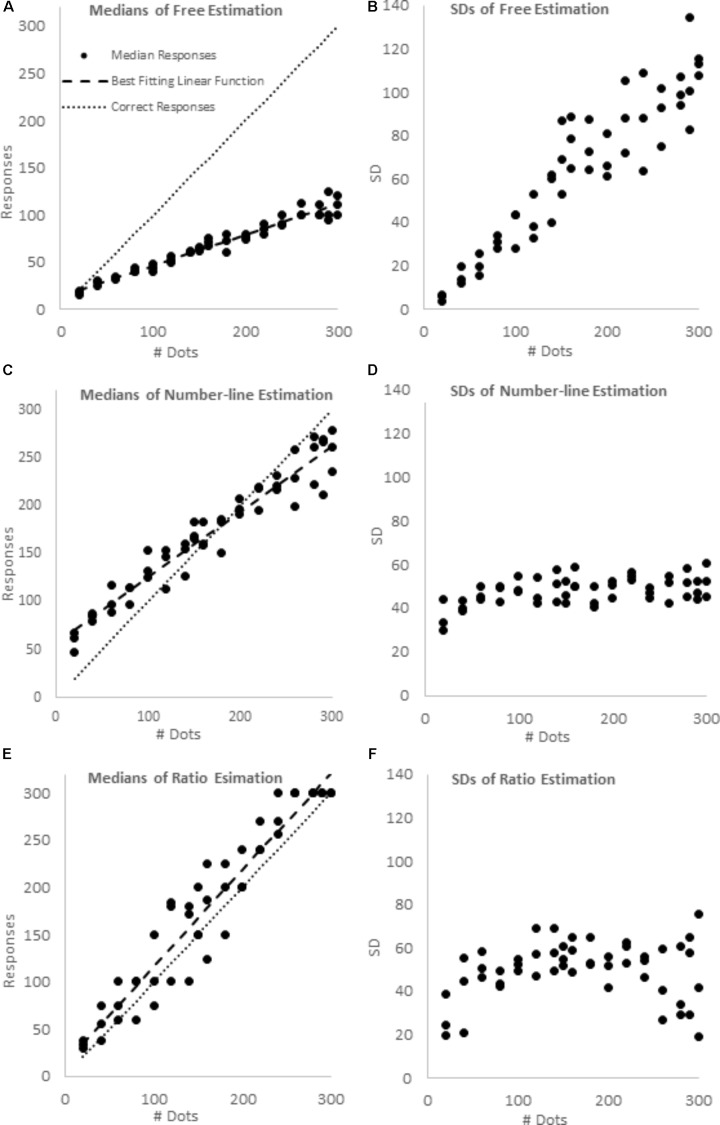
Median responses and *SD*s in the free estimation **(A,B)**, number-line estimation **(C,D)** and ratio estimation blocks **(E,F)**.

For each of the 51 stimuli (the 17 magnitudes in the three format) in each of the three blocks, we found the participants’ median responses, and the standard deviation of those responses. Plots of these data are presented in **Figure 4**. We fit the median responses to four different models:

$$\begin{array}{c} \mathrm{Linear}:\;\mathrm{median}=\mathrm{B*stimulus}+C\\ \mathrm{Logarithmic}:\;\mathrm{median}=\mathrm{B*ln}(\mathrm{stimulus})+C\\ \mathrm{Power}:\;\mathrm{median}={\mathrm{C*stimulus}}^{B}\\ \mathrm{One-cycle Cyclical Power Model}:\;\mathrm{median}=({\mathrm{stimulus}}^{B}/\\ ({\mathrm{stimulus}}^{B}+{\left(\mathrm{Range}-\mathrm{stimulus}\right)}^{B}))*\mathrm{Range} \end{array}$$

For consistency, all models were fit by minimizing the sum of squares distance to the predicted value, and all *R*^2^s were calculated as 1 – (Residual Sum of Squares)/(Corrected Sum of Squares). Parameters B and C were allowed to vary freely in all models. The 1-cycle cyclical power model did not include a C parameter, but rather included a Range parameter, which indicates the range of values over which responses may be given. The 1-cycle model was run both with Range fixed at 300, and with Range allowed to vary, but constrained to be greater than or equal to the maximum median value in the data set. We utilized the nonlinear regression function of SPSS version 21 to conduct these analyses. A linear regression was also run on the standard deviations. Regression results are presented in **Table 1**.

**Table 1:** Various regressions on median estimates and linear regressions on standard deviations for the free estimation, number-line estimation, and ratio estimation tasks.

Model	Free estimation	Number-line estimation	Ratio estimation
**Linear**			
B (*SE*)	0.327 (0.009)	0.683 (0.026)	1.020 (0.042)
C (*SE*)	13.708 (1.784)	57.210 (4.983)	14.573 (7.845)
*R*^2^	0.961	0.933	0.926
**Log**			
B (*SE*)	36.607 (2.113)	77.081 (4.613)	113.414 (7.637)
C (*SE*)	−111.437 (10.497)	−207.444 (22.915)	−372.217 (37.937)
*R*^2^	0.862	0.853	0.821
**Power**			
B (*SE*)	0.758 (0.030)	0.602 (0.030)	0.900 (0.051)
C (*SE*)	1.452 (0.231)	8.161 (1.289)	1.869 (0.512)
*R*^2^	0.960	0.930	0.926
**1-cycle cyclical power model, variable Range**			
B (*SE*)	N/A	0.603 (0.028)	1.125 (0.108)
Range (*SE*)	N/A	368.188 (14.896)	300.000 (19.005)
*R*^2^	N/A	0.934	0.891
**1-Cycle cyclical power model, range fixed at 300**			
B (*SE*)	N/A	0.581 (0.041)	1.125 (0.107)
*R*^2^	N/A	0.879	0.891
**Standard deviations**			
B (*SE*)	0.364 (0.020)	0.033 (0.009)	0.018 (0.021)
C (*SE*)	2.765 (3.803)	42.384 (1.705)	46.496 (4.006)
*R*^2^	0.869	0.212	0.015

### Regressions

As predicted, only the free-estimation task showed scalar variability (see **Table 1** and **Figure 4**). Indeed, set size accounted for over 86% of the variance in SD for the free estimation task, but less than 22% of the variance in SD for the number-line task, and less than 2% of the variance in SD for the ratio estimation task. In the Number-line estimation trials, SD had little relationship with the stimulus, and in the ratio estimation trials, SDs appear lowest for the extreme proportions of 0 and 1, and to peaknear 0.5.

Participants’ median responses appeared to increase linearly with stimulus magnitudes in all three conditions (see **Table 1** and **Figure 4**). Indeed, for all three blocks, the linear model was a better a fit than the logarithmic model and as good a fit as the standard power model. However, the ratio estimation and number-line estimation tasks were also well fit by cyclical power models, whereas a cyclical power model could not be fit to the free estimation task. Free estimation was the least accurate (Linear regression: slope = 0.327, intercept = 13.708), with responses consistently ∼1/3 of the true value, and ratio estimation was the most accurate (Linear regression: slope = 1.020, intercept = 14.573), with responses quite near the true values. Number-line estimation had intermediate accuracy (Linear regression: slope = 0.683, intercept = 57.210). As would be predicted by a cyclical power model, median number-line estimates were overly high below the midpoint of the range, relatively accurate near the midpoint, and too low above the midpoint. We confirmed that this over- then underestimation pattern was significant using binomial tests. For smaller arrays (i.e., 20, 40, 60, 80, and 100 dot arrays in each of the three formats) 15 out of 15 median estimates were greater than the stimulus values (*p* < 0.001). For larger arrays (i.e., 200, 220, 240, 260, 280, 290, and 300 dot arrays in each of the 3 formats) 20 out of 21 median estimates were less than the stimulus values (*p* < 0.001). However, the high and low endpoints failed to converge toward the anchors as we had predicted based on the cyclical power model.

## Discussion

Our results showed that task differences did in fact lead to vast differences in participants’ abilities to make accurate estimates from ANS-processed stimuli. We found that free estimation yielded underestimates throughout the tested range. In contrast, number-line estimations first over- and then underestimated the size of the stimuli, though via a shallow linear slope as opposed to the predicted cyclical power model. Finally, performance on ratio estimation tasks was quite accurate. Indeed, ratio estimation yielded an unbiased linear map to symbolic number, whereas both the free and number-line estimation tasks yielded biased maps. Further, only the free estimation task exhibited scalar variability. These differences emerged even though all three tasks featured stimuli that current theory would suggest are processed by the ANS. Such results would not be expected given the assumption that understanding of symbolic numbers is based on a direct mapping between number symbols and ANS-processed numerosities. These findings have implications for theories regarding the degree to which ANS-based estimation might serve as a good foundation for grounding symbolic number magnitudes.

### Implications for Mapping

#### Free Underestimation

Free estimations of dot arrays – a prototypical ANS task – led to considerable underestimates of the numerosities of the arrays, yielding the least accurate mappings of the three task formats. This is consistent with findings in prior literature (e.g., Indow and Ida, 1977; Izard and Dehaene, 2008; Crollen et al., 2011). Indeed, to our knowledge, free estimation of dot arrays has only proven accurate in three specialized situations: The first situation involves numbers in the subitizing range (up to ∼4–5 objects), which recruits the object tracking system (e.g., Chesney and Haladjian, 2011). Second, free estimation for numerosities between 4 and 8 dots are also accurate on average, although estimates are less precise than in the subitizing range (e.g., Taves, 1941; Kaufman et al., 1949). In the third instance, some have found that free estimation, although not precise, is accurate on average, with larger arrays when feedback is given after every single trial to allow calibration (Minturn and Reese, 1951). However, Izard and Dehaene (2008) showed that this calibration can easily be thrown off by a single instance of inaccurate feedback.

This poses considerable difficulties for accounts that argue that the ANS-based magnitude perception serves as a ground for specific numbers. Given the failure of free estimation to facilitate accurate maps between numbers and their nonsymbolic analogs, it makes sense to question whether the ANS can be used to ground number symbols in a direct 1-to-1 fashion. For example, presuming that the ANS response to an array of 20 dots could serve as a stable referent for the symbol “20” seems untenable given the demonstrated inaccuracy of free estimation. This is not to say that we should abandon the ANS-as-ground position entirely. Rather, we believe it necessary to re-examine how ANS magnitudes and symbolic numbers might be linked. The current data may offer some insight into how this might be accomplished.

Performance on the free estimation task was very well fit (*R*^2^ = 0.961) by a linear function with a slope of 0.327. Thus, although inaccurate, participants were quite reliable in their underestimation; they underestimated values at a consistent proportion of about 1/3. Of note, this particular underestimation yielded an estimate range with a maximum of approximately 100, even though the maximum array size was 300 dots. The large discrepancy is quite interesting, and we speculate that the value 100 may have a certain cultural status of being a default “large number.” This would explain why participants should happen to scale their responses so that the upper limit would be approximately 100. Given that prior research clearly demonstrates that adults can scale subsequent responses against a standard value (Izard and Dehaene, 2008; Thompson and Siegler, 2010), it is plausible that the 1/3 slope observed here was the result of “auto-scaling,” whereby participants assumed that the largest dot-set had 100 dots and scaled the remaining responses accordingly.

#### The Relational ANS

Although estimation patterns for all three tasks approximated linearity, ratio tasks clearly yielded the most accurate estimates. Median estimates were extremely well fit by a linear model with a slope of one and an intercept that was statistically equivalent to zero. Even the power model fit for ratio tasks yielded a Stevens’ exponent of 0.9, indicating a curve that is very close linear. Considering this result in light of prior research showing that people can make proportion judgments cross-modally with great accuracy (Matthews and Chesney, 2015), this offers an intriguing possibility for grounding unfamiliar number symbols: Perhaps one way to gain an intuitive understanding for the magnitude of an unfamiliar number symbol is to start with a known number symbol and to use a cross-format proportion to convey how large the unfamiliar number is compared to the familiar number (see also Leibovich et al., 2016).

Chesney and Matthews (2013) found results consistent with this using number lines. They had undergraduates perform a number line estimation task using a line that extended from 0 to 0.999 × 10^4.5^. Participants were unfamiliar with the magnitude of 0.999 × 10^4.5^ (i.e., 31,591) and performed poorly until given the hint that 16,000 was roughly halfway along the number line. This intervention greatly improved performance. Participants used cross format proportion matching (Barth and Paladino, 2011; Sidney et al., 2017) to map the source ratio – the line segments’ lengths – to the target ratio – the symbolic numbers. Thus they began to correctly treat 0.999 × 10^4.5^ as roughly twice as large as 16,000, or about 32,000. The unfamiliar symbol gained meaning. A similar process can be used to map symbolic to nonsymbolic ratios more generally. For example, if a child watches her grandmother mapping 8 grapes to a “handful” in a recipe, and later saw 16 grapes being mapped to a “cup,” she could determine that the ratio of a “handful” to a “cup” was about 1:2, and use this knowledge in deciphering quantities in future recipes.

This process might be used by children learning symbolic numbers. If they observe a set of 25 dots being referred to as “20” and a set of 50 dots being referred to as “40” – such dot arrays are often underestimated (Taves, 1941; Izard and Dehaene, 2008; Crollen et al., 2011) and can even be purposefully mapped to larger or smaller values with inducers (Izard and Dehaene, 2008) – they can learn that the ratio of “20” to “40” is 1:2. The observed symbolic number to nonsymbolic numerosity map might be biased, but the nonsymbolic ratio is maintained. Such enumeration biases would be immaterial if relational mapping is the primary mechanism supporting the link between symbolic and nonsymbolic quantities. Moreover, if a system of ratios between symbols is known (e.g., “5” is half “10,” “10,” is half “20,” “20” is half “40”), and at least one of the symbols is accurately mapped (e.g., five dots is “5”) then a sense of scale for the other mapped symbols can propagate forward. Thus, it may be an approximate sense of proportion that drives the link between ANS estimation and symbolic number, rather than a direct correspondence between a symbolic number and a specific ANS magnitude. This perceptually based ratio sense would have limited utility compared to exact symbolic representations (e.g., one can symbolically represent 300/500 and 301/500, but one is unlikely to distinguish between their nonsymbolic instantiations) but all such perceptually based processes are necessarily limited in this sense.

Although this account is speculative, it is quite consistent with psychophysical accounts of how ANS-based comparison is processed. Indeed, as Sidney et al. (2017) observed, Weber’s law is fundamentally parameterized in terms of ratios, which means that existing conceptions of the ANS are largely compatible with viewing the system as inherently relational (cf., McCrink and Spelke, 2010; McCrink et al., 2013). This viewpoint essentially recapitulates Birnbaum and Veit (1974) observation that differences and ratios are in some sense mathematically equivalent in the logarithmically transformed space of perception, given that a log-transformed ratio yields a subtraction (i.e., log(x/y) = log(x) – log(y). We do note that work remains to be done to square this relational conception with neuroscientific evidence of numerosity specific neurons (e.g., Nieder et al., 2002; Diester and Nieder, 2007). That said, the mathematics of the dominant model is incontrovertible, so a relational conception of the ANS should not be easily dismissed.

The relational view of the ANS may suggest that two numerosities are better than one when it comes to facilitating maps to number symbols. Using two numerosities when mapping ANS magnitude to symbolic numbers solves a perennial problem with free estimations – specifically the vast individual differences in these estimates. Importantly, ratio perception establishes a correspondence among multiple instantiations of the same ratio, e.g., 10/15, 20/30, 50/75, etc. Thus, there is an inherent calibration for ratio judgments that may largely circumvent idiosyncratic scaling seen in single judgments. These observations converge with emerging theories about how ratio might be used to establish maps from perception of continuous magnitudes to specific numbers – as argued, for instance by Sidney et al.’s (2017) commentary on Leibovich et al. (2017) generalized magnitude system theory. They also converge with theories positing that ratio might be the preferred format for equating perceived magnitudes across different modalities (Balci and Gallistel, 2006; Bonn and Cantlon, 2017). All combined, we interpret the data as suggesting that the ANS is perhaps best understood as a system that perceives relations between numerosities, and as such may be more accurate when used to assess ratios as opposed to whole numbers. Future research should investigate this possibility.

### Limitations and Future Directions

#### Memory Issues in Number-Line Estimation

As noted above, our prediction that performance in the number-line estimation task would be characterized by a cyclical power model was not fully supported: although median estimates were overestimated below the midpoint of the range, relatively accurate near the midpoint, and underestimated above the midpoint, the high and low endpoints failed to converge toward the anchors. This may have been due to the speeded presentation protocol we used in order to ensure that participants could not count individual dots. As soon as the stimuli disappeared from view, they had to be maintained in memory and were thus subject to decay. Although this applies to all three tasks, this speed component may have specifically complicated the number-line task. Free estimation and ratio estimation tasks like those used here are typically conceived of as involving relatively direct estimation. However, the proportion judgment model conceived of by Spence (1990) and Hollands and Dyre (2000) involves explicit strategies whereby the observer pegs landmark values that result from segmenting the range (e.g., into halves or fourths) and subsequently estimates the remaining distance between the stimulus and the reference point. Memory decay may thus have more substantially impacted the bounded-estimation process than the other two tasks. In future work, we will compare performance in speeded and unspeeded conditions. We will also investigate potential differences in performance that might be induced by instructions focusing on an explicit ratio match versus instructions that focus on the landmark-based proportion judgment of the Spence (1990) model.

#### Free Estimation, Linear Compression

One interesting result specific to the free estimation task was that, although participants consistently underestimated the dot array magnitudes, their estimates did not appear compressive in the traditional sense that they were better fit by a logarithmic or power function than a linear function, or that the proportion of underestimation became greater as the set size increased. Rather, the portion of underestimation remained constant. This linear performance is more typical of sequentially presented stimuli than the simultaneous presentation we used here (Taves, 1941; Meck and Church, 1983; Cordes et al., 2001; Izard and Dehaene, 2008; Crollen et al., 2011). While this may have been an idiosyncrasy of our data set, it is possible that this was due to our choice of stimuli. Our smallest value, 20, was well above the subitizing range (∼4, Taves, 1941; Chesney and Haladjian, 2011). Numerosity estimates are known to be quite accurate when people subitize (Taves, 1941; Chesney and Haladjian, 2011). There also appears to be a benefit to accuracy when estimating values immediately above this range (e.g., 6, 7, 8; Taves, 1941; Kaufman et al., 1949), possibly due to subitizing based strategies: at the very least, these values would be known to be greater than ∼4. Our results show that people are linear with a slope less than 1 for larger values. Including both accurately assessed, subitizing-influenced low number values and underestimated higher values in a stimulus set would yield bi-linear performance. Regressions comparing compressive power or log functions to (mono-)linear functions for such bi-linear data would favor the compressive functions. Further work is needed to assess if (mono-) linear rather than compressive estimation patterns are typically seen when values that may be aided by subitizing strategies are excluded from consideration.

## Conclusion

There are three main takeaways from these results. First, number-line estimation tasks appear to have limited utility in investigating either the ANS or the mapping between the ANS and symbolic numbers. These tasks do not yield the classic error patterns (i.e., scalar variability) seen in ANS estimation, and the functional form of performance on line-estimation tasks does not necessarily parallel the functional form of individuals’ underlying magnitude representations. The use of nonsymbolic stimuli does not overcome these limitations. Second, the underestimation in the free-estimation task, particularly relative to the accurate performance on the proportion judgments task, is problematic for theories that propose a direct mapping between symbolic numbers and ANS estimation of specific nonsymbolic magnitudes. Third, we suggest that a system that uses a sense of ratio to link symbolic numbers to ANS-perceived magnitudes may overcome these difficulties. Future research is needed to address these possibilities.

## Ethics Statement

This study was carried out in accordance with the recommendations regarding human subjects research by the Internal Review Board (IRB) of the University of Notre Dame. The protocol was approved by the IRB of the University of Notre Dame. All subjects gave written informed consent in accordance with the Declaration of Helsinki.

## Author Contributions

All authors listed have made a substantial, direct and intellectual contribution to the work, and approved it for publication.

## Conflict of Interest Statement

The authors declare that the research was conducted in the absence of any commercial or financial relationships that could be construed as a potential conflict of interest.
